# Human xenograft models as useful tools to assess the potential of novel therapeutics in prostate cancer

**DOI:** 10.1038/sj.bjc.6604822

**Published:** 2008-12-16

**Authors:** W M van Weerden, C Bangma, R de Wit

**Affiliations:** 1 Department of Urology, Josephine Nefkens Institute, Erasmus MC, Rotterdam, The Netherlands; 2 Department of Medical Oncology, Erasmus MC, Rotterdam, The Netherlands

**Keywords:** hormone refractory prostate cancer, xenografts, novel therapeutics, PSA biomarker

## Abstract

With docetaxel as effective chemotherapy for hormone refractory prostate cancer (HRPC), the number of new treatment combinations for HRPC is expanding demanding a fast-track screening system. This review elaborates on the use of xenograft models to select the most promising combination therapies for entering into phase II clinical trials.

## 

### The shifting paradigm of chemotherapy in hormone refractory prostate cancer

During the past decades the systemic treatment of most solid cancers has made multiple developmental steps forward. Today, in most cancers, first, second and even subsequent lines of therapy are available, which in addition to relieving symptoms and delaying disease progression may prolong survival by months or years, even in the setting of overt metastatic disease. Although the introduction of new therapeutic agents in areas such as breast cancer, colorectal cancer and lung cancer has been rather successful in many aspects, the advent of effective chemotherapy in hormone refractory prostate cancer (HRPC), unfortunately, has been one of the exceptions. During the 1980s and 1990s, numerous single agent chemotherapy trials in HRPC consistently showed poor overall response rates, without any trend towards improvement in survival (Yagoda and Petrylak, 1993). Based on this apparent lack of activity of chemotherapy in HRPC, and the common perception that this elderly patient population was, in general, too fragile to receive such treatment, patients with HRPC have not been routinely treated with chemotherapy for many years and few investigators pursued clinical trials in this field. In 2004, this paradigm shifted considerably following the results of two independent phase III studies that showed robust survival improvement as well as improved symptom relief and improved quality of life, by the use of docetaxel-based chemotherapy as compared with the standard regimen of mitoxantrone plus low-dose prednisone (Petrylak *et al*, 2004; Tannock *et al*, 2004). Overall survival (the primary end point) was superior for the docetaxel groups compared with mitoxantrone-treated patients. In the TAX327 study comparing docetaxel every 3 weeks, docetaxel weekly and mitoxantrone every 3 weeks, all arms with low-dose prednisone, the median duration of survival was 19.3, 17.8 and 16.3 months, respectively (Berthold *et al*, 2008). Also, significantly more patients obtained a pain response and improvement of their quality of life. The SWOG 99-16 study showed a 2-month survival benefit of the docetaxel plus estramustine arm (17.5 months) as compared with the standard therapy of mitoxantrone plus prednisone (15.6 months). The addition of estramustine to docetaxel, increased gastrointestinal as well as cardiovascular toxicity. As a result, in 2004 docetaxel every 3 weeks plus prednisone became the standard treatment recommendation for patients with HRPC as approved by the American Food an Drug Administration (FDA) and European Agency for the Evaluation of Medical Products (EMEA) (De Wit, 2005).

With the establishment of this new standard reference treatment for HRPC, this opened the clinical investigational programme of HRPC for a myriad of phase II and phase III trial options. Today, at least seven large phase III randomised trials are in progress, all investigating the potential of adding a new agent to docetaxel every 3 weeks plus prednisone; bevacizumab (CALGB), atrasentan (SWOG), high-dose pulse calcitriol, DN 101 (Novacea), risedronic acid (Netherlands-Norwegian study), ZD4054 (AstraZeneca), GVAX (Cel Genesys) and VEGF trap (Sanofi-Aventis). Many more options have a similar good rationale, but have not passed the drawing board of the Pharmaceutical Industries, due to the current competition and the risk of being faced with a new standard therapy during trial conduct. As a result, both industries and investigators hesitate to embark on new large clinical trials at this point of time.

### Surrogate end points in HRPC

A particular additional problem with clinical research in HRPC is the increasing concern with the usefulness of surrogate end points (e.g. biomarkers). With the piling number of options for phase III trials of HRPC, and overall survival being the only accepted end point, such randomised studies are large and lengthy. The need for intermediate end points to evaluate treatment effects early in time is urgently required. However, alternative end points such as progression free survival, even if confined to clinical criteria for progression, has shown to poorly correlate with overall survival (Di Lorenzo *et al*, 2007). Serial monitoring of prostate specific antigen (PSA) is often used as an indicator of treatment response, but a direct correlation with tumour load and/or outcome has never been made in the clinical setting (Hadaschik *et al*, 2007). Although PSA response may be considered a modest surrogate measure for survival, this has hardly been tested in the setting of molecular-targeted agents. Several other (serum) markers have been proposed as additional or alternative biomarkers or surrogate end points, but so far these markers are in experimental phase and lack clinical validation.

### Human xenografts for fast-track testing of new therapies for HRPC

To increase the speed of selection of the large amount of new options for HRPC, an alternative strategy is proposed that makes use of human xenograft models of PC as a pre-screening system (Schröder *et al*, 2000). Such a xenograft-based pre-screen would enable a relatively fast selection of the best performing compound, of new combinations and their optimal sequence, as well as their potential superiority towards docetaxel treatment. Also, such preclinical studies would allow for validation of biomarkers, such as PSA, for their use in subsequent phase II clinical studies. In xenograft models, circulating levels of PSA can be directly correlated to tumour burden, allowing validation of the relationship between PSA and tumour response under therapy (Limpens *et al*, 2006). This is essential as compounds may interfere with the regulation of PSA production and/or secretion without affecting tumour growth, consequently resulting in PSA responses that do not reflect tumour responses.

## Preclinical models of human PC

### The establishment of PC xenografts and cell lines

The PC research field has long been hampered by the limited availability of representative model systems. The ‘classical’ models are the androgen-independent PC3 and DU145 cell lines and the androgen-responsive LNCaP cell line (Van Bokhoven *et al*, 2003). PC3 and DU145 lack expression of androgen receptor and PSA, characteristics known to be present in HRPC (Ruizeveld de Winter *et al*, 1994), and therefore poorly reflect HRPC. Although more clinically relevant, the androgen responsive, PSA-secreting LNCaP cell line has its limitation in its poor *in vivo* tumorigenicity and in an aberrant response to antiandrogens due to a point mutation in the ligand binding domain of the androgen receptor that renders the cells sensitive to not only androgens, but also to other hormones such as progesterone and oestrogen, as well as to antiandrogens such as hydroxyflutamide (Veldscholte *et al*, 1992).

The number of human-derived xenografts have been very few and only in the early 1990s were we able to establish significant number of human PC xenograft models (Van Weerden *et al*, 1996). This success has triggered other groups, primarily in the United States, to intensify their efforts resulting in a further extension of human-derived PC xenografts such as the CWR, MDA Pca, LuCaP and LAPC series of xenografts established by different groups in the United States (Table 1). All these xenograft models are made available to other researchers (Bosland *et al*, 1996, Navone *et al*, 1999).The development of permanent cell lines from PC xenografts has been rather complicated, being successful only for the PC346 xenograft, resulting in the PC346C cell line (Marques *et al*, 2006). In recent years, a small number of xenograft-derived cell lines have been established by other research groups as well. These novel cell lines PC346C, 22Rv1, CWR-R1, DuCaP, LAPC-4, MDA Pca1, MDA Pca 2a, MDA Pca 2b and VCaP have all been karyotyped and compared in an extensive study by van Bokhoven *et al* (2003). In Rotterdam, further *in vivo* and *in vitro* selection procedures have resulted in additional PC346 sublines with different (molecular) characteristics that may be related to the various stages of PC progression (Marques *et al*, 2005, 2006). The present set of xenograft models and cell lines represent the various disease stages of human PC and strongly add to the options for preclinical testing.

Xenograft models are very well suited for molecular studies as they consist of pure human tumour tissue without contamination of human normal prostate tissue, which is always present in patient samples. Xenografts have been instrumental to evaluate the extent of abnormal genetic changes and gene profiles in human PC by various research groups. Genomic characterisation shows that specific genomic abnormalities that have been detected in the PC patient population, such as mutations in the *PTEN* suppressor gene and genetic alterations in specific genes are also prevalent in the xenograft panel (Vlietstra *et al*, 1998; Maki *et al*, 2007). Moreover, xenograft studies revealed TMPRSS2-erg translocations as well as alternative TMPRSS2-ETV4 translocations that were not yet detected in men (Hermans *et al*, 2006, 2008). Knowledge on the genomic profile of xenografts is crucial also for selecting the most appropriate preclinical model, especially in the new era of targeted therapies that require model systems with a specific molecular expression profile that mimic a specific disease stage or patient group.

### The orthotopic PC xenograft model

The majority of preclinical studies are being performed using subcutaneous injected tumour cells or xenografts. With the establishment of several new human PC cell lines from xenografts, orthotopic injections of PC346C, LAPC4 and LuCaP cell lines into the mouse prostate have became an attractive alternative for the traditional subcutaneous xenograft model. This especially holds true for the evaluation of targeted therapies where expression of the tissue-specific target is essential and may well be influenced by the implantation environment.

**Figure 1:**
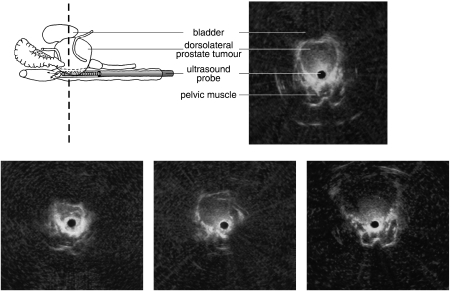
Transrectal ultrasonography to monitor orthotopic tumour growth in the mouse prostate. PC346C human prostate cancer cells were injected into the dorsolateral lobe of the mouse prostate and prostate volume was imaged longitudinally Kraaij *et al* (2002).

A drawback of orthotopic transplantation in the mouse prostate is the difficulty of monitoring tumour growth in time. In the case of PSA-producing cells, plasma PSA may be used as an indicator of tumour burden, although this approach would lack validation of circulating PSA levels to actual tumour load (Thalmann *et al*, 1996). To allow longitudinal studies in individual mice to evaluate treatment efficacy, a three-dimensional ultrasound micro-imaging technique was developed that allows us to frequently monitor prostate (tumour) volume (Kraaij *et al*, 2002). For many years, this micro-imaging technique using transrectal ultrasonography is a validated and routine procedure to monitor prostate volume in all our orthotopic studies (Figure 1).

### Tumour characteristics of PC xenografts

There is a general feeling that xenograft data have a poor prediction for human responses in clinical trials. As the three ‘classical’ cell line models, PC3, DU145 and LNCaP, are strongly overrepresented in these types of studies, this feeling may well relate to the inappropriate choice of the model system. With the present generation of xenograft models and the detailed knowledge of their genomic profiling, it is believed that human xenografts are very powerful tools to investigate compound efficacy, and to define compound specificity (in case of a target-specific agent) or identify (in case no target is known) the mechanism of action. Moreover, evaluation of combination therapies and their most optimal sequence of administration are important issues and xenograft models will constitute a very valuable asset to select for potential clinical efficacy.

To perform relevant preclinical studies, the choice of the most appropriate test model is essential and depends largely on the patient group for which the treatment is defined and, in relation to that, the expression of the target of interest. Along with extensive knowledge of the gene and/or protein expression profiles, xenograft responses to first-line treatment for PC, hormonal ablation therapy, and to the second-line treatment, docetaxel, are additional selection criteria.

### Androgen responsiveness

Hormonal ablation treatment, either by surgery or chemically, have been the hallmark of treatment for advanced PC since the first studies of Huggins and Hodges in the early 1940s. Since then, androgen responsiveness and later androgen receptor characteristics as well as PSA expression have been the most important determinants for models of PC (see Table 1). Most tumours from PC patients that no longer respond to endocrine therapy have retained a functional androgen receptor with often higher levels than in primary tumours (Ruizeveld de Winter *et al*, 1994). Approximately 30% of recurrent tumours show amplification of the androgen receptor gene with an estimated 10–30% of antiandrogen-treated patients having a mutated androgen receptor (Linja and Visakorpi, 2004). These data strongly suggest that the androgen receptor is still involved in growth regulation of HRPC and that the presently available antiandrogens (such as flutamide and bicalutamide) are not capable of blocking this activity in the HRPC phase of PC. With the increasing knowledge on androgen receptor activation mechanisms, novel generation androgen receptor-targeted molecules are being developed that aim to overcome this resistance by acting better than or differently from the currently available, conventional androgen receptor antagonists. Also, new efforts are directed towards specific blocking of enzymes involved in (intratumoural) steroidogenesis as studies have indicated that HRPC may have the potential capacity to produce its own androgens (Montgomery *et al*, 2008). The PC346 xenograft system (Marques *et al*, 2006) as well as some of the CWR22, LuCAP and LAPC xenograft lines show progressive, androgen-independent growth after androgen ablation of tumour-bearing mice. These resistant, androgen-independent sublines are valuable assets to study androgen-resistant molecular pathways (Hendriksen *et al*, 2006). We also established a set of antiandrogen (flutamide) resistant PC346C cell lines that mimic the clinical situation of HRPC. This unique cell line panel that can also be grown as xenografts, reflects the various mechanisms of androgen resistance that are also observed in patients, including mutation, overexpression and downregulation of the androgen receptor (Marques *et al*, 2005). Clearly, such cell lines and xenograft models are instrumental for *in vitro* and *in vivo* testing of novel antiandrogens.

### Response to docetaxel

**Figure 2:**
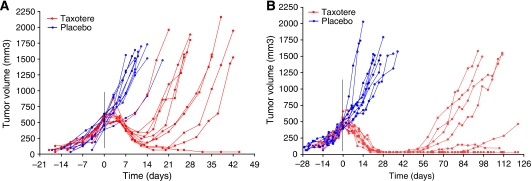
Response of the human prostate xenografts PC-339 (**A**) and PC374 (**B**) to docetaxel treatment. Docetaxel was administered as one bolus injection i.p. of 33 mg kg^−1^ to mice on day 0 when tumours reached a size of approximately 500 mm^3^. Tumour volume was recorded by transrectal ultrasound (**A**) and calipers measurements (**B**). Mean growth curves (mean±s.e.m.) represent data from six animals per group.

With the establishment of docetaxel as effective treatment for HRPC, the need for representative test systems to compare combination strategies became relevant and information regarding docetaxel responses of the various cell lines and xenograft models has become an additional selection parameter. In a series of studies we tested the sensitivity of our xenograft models to one single injection of docetaxel (33 mg kg^−1^, i.p.). Of the seven xenografts that we have tested so far, all responded with tumour growth reduction, with none showing initial resistance to docetaxel. The level of response was variable ranging from 50% volume reduction in the androgen-dependent PC82 tumours to complete remission of the androgen-independent PC374 tumours. Duration of response ranged from 14 to 30 days, with an exception for PC374 that showed long-term individual responses for up to 100 days (Figure 2). An additional injection of docetaxel showed that relapsed tumours remained sensitive to docetaxel treatment. Also, injections with a lower dose of docetaxel (17 mg kg^−1^, i.p.) resulted in similar response patterns typical for a particular xenograft although the effect was less pronounced with regard to tumour inhibition and duration of response (data not shown). The variability of response of these xenografts to docetaxel treatment reflects the response profiles that are also observed in the clinic (Azim and Mok, 2008). To improve magnitude and duration of clinical responses to docetaxel treatment, new combination therapies have been proposed. The present xenograft data is currently providing a solid basis to test these new combinations for their efficacy to prolong and sustain the initial docetaxel effect in HRPC.

## Preclinical PSA validation and PSA-based phase II trials

The number of compounds and combination options to be tested for HRPC are rapidly increasing and demand a fast-track test system to improve the entrance of promising compounds into clinical trials. In Rotterdam, we have evaluated a strategy of parallel testing of new compounds using preclinical xenograft studies and experimental, PSA-based, short-term clinical phase II studies (Kranse *et al*, 2005; Schroder *et al*, 2005). The xenograft studies were used to confirm efficacy of the compound as well as to validate PSA as a surrogate biomarker for therapy response. For the PSA-based phase II studies hormone naive patients were selected who were treated by local therapy (radical prostatectomy or radiation therapy) and who were confronted with rising PSA of unknown origin. Patients were randomised into a supplement or placebo group. The study was double-blind and had a crossover design with a wash-out period in between both treatment periods. Changes in PSA kinetics were used as parameters. This set-up allows for concise and short-term studies with a duration of less than 6 months without loss of statistical power (Schröder *et al*, 2000).

In one of the first studies that used this strategy, the effect of the selective EGFR tyrosine kinase inhibitor gefitinib (ZD1839, Iressa™) as monotherapy was evaluated. In contrast to similar studies with the CWR22 xenograft (Sirotnak *et al*, 2002), we were unable to show a tumour inhibitory effect of gefitinib (daily oral doses of 50, 100 and 200 mg kg^−1^) on orthotopic tumour growth or PSA release of PC346C, whether given to established tumours (>200 mm^3^) or directly after tumour inoculation (Limpens *et al*, 2005). However, although not effective, Iressa did not preferentially interfere with PSA release and, thus, could be considered as a surrogate biomarker for tumour response under Iressa therapy in the clinical setting. Indeed, additional clinical studies confirmed the limited activity of gefitinib as monotherapy in PC (Schröder, 2003). Further molecular studies in our PC346 cell line model revealed that, although gefitinib effectively blocked EGFR phosphorylation in PC cell lines, it failed to decrease the constitutively high Akt activity that is present in those PC lines that have a non-functional *Pten* (PC3, LNCaP, PC346C). As Pten loss is a frequent event in PC, this may well explain the low sensitivity of PC cells to gefitinib.

A second series of experiments that used this screening strategy were based on the evaluation of dietary supplements to delay PC progression. Differences in incidence rates of clinical PC with low incidence rates in Asian countries and high rates in Europe and the United States, have since long suggested a role of life style factors such as diet, in PC progression. Epidemiological data and intervention studies have further triggered a number of large-scale prevention and intervention studies. Also, experimental studies using both human PC cell lines and xenografts have been performed to elucidate mechanisms of chemopreventive action of a variety of dietary agents. As the number of supplement combinations and dosages are nearly infinite, our selection strategy using both PC xenografts and PSA-based clinical phase II study was used to identify the most potential combinations. Two randomised double-blind and placebo-controlled PSA-based phase II studies have been performed in Rotterdam with two different oral supplements constituted of several dietary nutrients including soy isoflavones, lycopene, selenium and antioxidants. Both studies showed prolongation of PSA doubling time when patients received the supplement, suggesting reduction in tumour progression (Kranse *et al*, 2005; Schroder *et al*, 2005). In a third preclinical study, the antitumour effect of two dietary nutrients, synthetic lycopene and vitamin E, was tested. The experimental study revealed a significant inhibition of orthotopic PC346C tumour growth in those animals that had received low-dose lycopene plus low-dose vitamin E as compared with control animals Limpens *et al*, 2005). These tumour responses could be reproduced when using PSA as parameter, indicating no preferential effect of the supplement on PSA release. Based on this outcome, a PSA-based clinical phase II study is currently in progress in Germany.

## Conclusion

The introduction of docetaxel as effective treatment for HRPC has generated the need for fast-track screening to select new therapeutic agents and potential treatment combinations, thereby facilitating the proper design of phase II and phase III clinical trials. The generation of a substantial number of relevant PC xenografts and cell lines with stage-specific characteristics has significantly improved the potential application of preclinical models for testing of therapy efficacy. Docetaxel treatment of PC xenograft-bearing mice have shown that these xenograft responses mimic the clinical situation of HRPC and new combination therapies with docetaxel can now be evaluated in these xenografts. Such preclinical studies will also provide essential data on the relation between tumour volume and PSA responses allowing for the validation of PSA as potential surrogate marker in consecutive clinical studies. The concept of combining preclinical xenograft studies to PSA-based early phase II clinical trials allows for concise and relatively high-throughput selection system. Such a set-up has shown its value for the treatment of hormone naive patients and may also be applicable to improve the management of HRPC.

## Figures and Tables

**Table 1:** Human prostate cancer xenograft panel

**Name**	**Derived from**	**Androgen responsive**	**PSA**	**AR**	**Year Derived**
PC-82	Prostate	Yes	Yes	Yes	1977
PC-133	Bone	No	No	No	1981
PC-135	Prostate	No	No	No	1982
PC-EW	Prostate	Yes	Yes	Yes	1981
PC-295	LN	Yes	Yes	Yes	1991
PC-310	Prostate	Yes	Yes	Yes	1991
PC-324	TURP	No	No	No	1991
PC-329	Prostate	Yes	Yes	Yes	1991
PC-339	TURP	No	No	No	1991
PC-346	TURP	Yes	Yes	Yes	1991
PC-346I	PC-346	No	Yes	Mutant	1992
PC-346B	TURP	Yes	Yes	Yes	1991
PC-346BI	PC-346B	No	Yes	Yes	1992
PC-374	Skin	Yes	Yes	Yes	1992
TEN/12	Prostate	Yes	Yes	Yes	1985
LuCaP 23.1	Lymph node	Yes	Yes		1996
LuCaP 23.8	Lymph node	Yes	Yes		1996
LuCaP 23.12	Liver	Yes	Yes		1996
LuCaP 35	Lymph node	Yes	Yes	Yes	2003
LuCaP 35V	LuCaP 35	No			2003
LuCaP 49	Metastasis?	No	No	No	2002
LAPC-3	AI TURP	No	Yes/no	Yes	1999
LAPC-4	AI LN	Yes	Yes	Yes	1997
LAPC-9	AI Bone Met	Yes	Yes	Yes	2001
CWR22	AD Met	Yes	Yes	Mutant	1993
CWR21	AD Met	Yes	Yes	Yes	1993
CWR31	AD Met	Yes	Yes	Yes	1993
CWR91	AD Met	Yes	Yes	Yes	1993
MDA Pca-31	Liver	NA			1998
MDA Pca-40	Liver	NA			1998
MDA Pca-43	Adrenal	NA			1998
MDA Pca-44	Skin	NA			1998

AR=androgen receptor; LN=lymph node metastasis; MET=metastasis; NA=not applicable; PC=prostate cancer; PSA=prostate-specific antigen; TURP=transurethral resection of the prostate.
